# Injectable Xenogeneic Dental Pulp Decellularized Extracellular Matrix Hydrogel Promotes Functional Dental Pulp Regeneration

**DOI:** 10.3390/ijms242417483

**Published:** 2023-12-14

**Authors:** Shengmeng Yuan, Xueting Yang, Xiuting Wang, Jinlong Chen, Weidong Tian, Bo Yang

**Affiliations:** 1 State Key Laboratory of Oral Diseases, National Clinical Research Center for Oral Diseases, West China Hospital of Stomatology, Sichuan University, Chengdu 610041, China; yuansm1996@163.com (S.Y.); wxtfighting666@163.com (X.W.); jinlongchen@scu.edu.cn (J.C.); 2 National Engineering Laboratory for Oral Regenerative Medicine, Engineering Research Center of Oral Translational Medicine, Ministry of Education, West China Hospital of Stomatology, Sichuan University, Chengdu 610041, China; 3 Department of Oral and Maxillofacial Surgery, West China Hospital of Stomatology, Sichuan University, Chengdu 610041, China

**Keywords:** decellularized extracellular matrix (dECM) hydrogel, dental pulp stem cells, odontogenic differentiation, angiogenesis, dental pulp regeneration

## Abstract

The present challenge in dental pulp tissue engineering scaffold materials lies in the development of tissue-specific scaffolds that are conducive to an optimal regenerative microenvironment and capable of accommodating intricate root canal systems. This study utilized porcine dental pulp to derive the decellularized extracellular matrix (dECM) via appropriate decellularization protocols. The resultant dECM was dissolved in an acid pepsin solution to form dECM hydrogels. The analysis encompassed evaluating the microstructure and rheological properties of dECM hydrogels and evaluated their biological properties, including in vitro cell viability, proliferation, migration, tube formation, odontogenic, and neurogenic differentiation. Gelatin methacrylate (GelMA) hydrogel served as the control. Subsequently, hydrogels were injected into treated dentin matrix tubes and transplanted subcutaneously into nude mice to regenerate dental pulp tissue in vivo. The results showed that dECM hydrogels exhibited exceptional injectability and responsiveness to physiological temperature. It supported the survival, odontogenic, and neurogenic differentiation of dental pulp stem cells in a 3D culture setting. Moreover, it exhibited a superior ability to promote cell migration and angiogenesis compared to GelMA hydrogel in vitro. Additionally, the dECM hydrogel demonstrated the capability to regenerate pulp-like tissue with abundant blood vessels and a fully formed odontoblast-like cell layer in vivo. These findings highlight the potential of porcine dental pulp dECM hydrogel as a specialized scaffold material for dental pulp regeneration.

## 1. Introduction

Dental pulp disease is a prevalent infectious condition that significantly impacts human health. Different treatments can be used according to the inflammatory condition of the pulp and the degree of root development, such as pulp capping procedures, vital pulpotomy, pulp regenerative procedures, and root canal treatments [1,2,3]. Vital pulp therapies and regenerative endodontic procedures may help to avoid endodontic treatment, yet their effectiveness is mostly confined to limited cases and carries a less certain prognosis [4,5,6,7]. Conversely, conventional root canal treatments, suitable for most cases of pulp disease, aimed to extirpate inflamed or necrotic pulp tissue and substitute it with synthetic biomaterial, which can lead to tooth deactivation and possibly eventual tooth fracture [1,8,9,10]. The loss of sound and vital pulp poses a great challenge to long-term tooth survival and preservation. Fortunately, dental pulp tissue engineering holds promise in addressing these challenges.

Extensive research has highlighted dental pulp tissue engineering as one of the most promising approaches for achieving functional dental pulp regeneration [11,12,13]. However, several challenges remain, particularly concerning the development of bioactive materials containing intricate regenerative signals capable of establishing tissue-specific microenvironments conducive to dental pulp regeneration [14,15]. These challenges are further compounded by the narrow nature of the root canal environment where the dental pulp resides, coupled with the significant anatomical variations in root canal shapes [16,17], significantly complicating the development of scaffold materials. Currently, injectable hydrogels, such as alginate, chitosan, hyaluronic acid, collagen, gelatin methacrylate (GelMA), and complexes of natural polysaccharides and polypeptides, such as copolymers of sodium D-mannuronate and L-guluronate with gelatin [15,18,19,20,21], are considered suitable scaffolds for dental pulp tissue engineering, primarily due to their ability to conform to the complex shaping requirements of root canal systems and their potential to bind bioactive substances and transport stem cells [15,18,22]. Although these hydrogels boast a rich source and large-scale production capacity, they are unable to replicate the intricate components of the dental pulp extracellular matrix (ECM) and the bioactive molecules they carry, thus falling short in meeting the tissue-specific bioactivity demands crucial for effective dental pulp regeneration.

The decellularized extracellular matrix (dECM) has demonstrated its capacity to preserve tissue-specific biochemical and physical cues, offering structural support, adhesion sites, growth factors, and the ability to guide cell migration, organization, and stem cell differentiation, rendering it an optimal source for regenerative scaffolds [23,24,25,26]. Particularly, dECM hydrogels retain vital extracellular matrix proteins, such as collagen, gel at physiologically relevant temperatures and pH levels, assuming a nanofibrous architecture. They also exhibit excellent injectability and adjustable mechanical properties, significantly broadening the scope of dECM material applications in regenerative medicine [27,28,29,30]. To date, two types of dental pulp dECM hydrogels sourced from bovine and human origins have been reported, with preliminary evidence suggesting that these hydrogels support the adhesion, migration, proliferation, and odontoblastic, neurogenic, and angiogenic differentiation of dental pulp stem cells (DPSCs) in vitro [31,32]. These findings suggest that dental pulp dECM hydrogels hold promise as tissue-specific materials for dental pulp regeneration. Unfortunately, the specific effects of this kind of hydrogel on dental pulp regeneration in vivo have not been reported in subsequent experiments. This knowledge gap may be attributed to the limited availability and ethical constraints associated with human dental pulp tissue [32]. Furthermore, the reported bovine dental pulp dECM hydrogel has lost its injectability due to the complexities of the cross-linking process [31]. These challenges prompted our search for novel animal-derived dental pulp dECM materials.

Porcine-derived biomaterials are considered for human xenotransplantation to address the limitations associated with autologous or allogeneic materials. This preference is primarily due to their abundant sources without ethical constraints, relatively low production costs, and substantial genomic and physiological similarities to humans [33,34,35]. In 2006, Giovanna Orsini et al. introduced a biomaterial composed of cortical pig bone granules for maxillary sinus augmentation, demonstrating its favorable biocompatibility [36]. Additionally, the use of porcine or porcine dentin-derived bone graft materials in extraction sockets has shown promising outcomes in promoting alveolar ridge preservation and subsequent bone regeneration post-tooth extraction [37,38]. Despite the challenges of xenotransplantation, including the potential for recipient immune rejection and pathogen infection risks, various treatments such as decellularization, antigen removal, gamma radiation sterilization, and viral inactivation have substantially mitigated scaffold antigenicity and minimized graft-related infections as much as possible [39,40,41,42]. Currently, certain materials have advanced to translational applications. For example, the decellularized porcine small intestinal submucosa has found widespread application in the clinical reconstruction and repair of various tissue lesions [43]. Additionally, the well-known Geistlich Bio-Gide membrane, a resorbable collagen membrane derived from porcine sources, when combined with absorbable bone materials, has become a standard in guided bone and tissue regeneration procedures [44,45,46,47]. This widespread use indicates that biomaterials derived from porcine sources have evolved into a dependable option for human xenotransplantation. Previous studies have involved the preparation of porcine dental pulp dECM, confirming the retention of crucial ECM components and growth factors, including dentin matrix protein 1 (DMP-1), dentin sialoprotein (DSP), Collagen I, Collagen IV, laminin, fibronectin, transforming growth factor β1 (TGF-β1), and vascular endothelial growth factor (VEGF). These studies have demonstrated the capacity of these materials to induce odontogenic differentiation of stem cells, with some also providing preliminary evidence of their viability for in vivo dental pulp regeneration [48,49,50,51]. Regrettably, these studies solely employed porcine dental pulp dECM in solid form, which poses challenges in meeting the intricate shaping demands of complex root canal systems, significantly limiting its potential for clinical translation. Hence, there exists a pressing need to transform this material into a fluid hydrogel form capable of adapting to various shapes.

In this study, we developed a dECM hydrogel using porcine dental pulp as the primary material to obtain a microenvironment specific to the dental pulp. To evaluate the feasibility of application in complex root canal systems, we analyzed the rheological properties of dECM hydrogels. GelMA hydrogel served as the control. Furthermore, we investigated their effects on the biological behaviors of human DPSCs and implanted these cell-encapsulated hydrogels into a semi-orthotopic model using immunodeficient nude mice to assess the effectiveness of dental pulp regeneration in vivo. Our findings highlight the favorable injectability of porcine dental pulp dECM hydrogels and their potential to stimulate functional dental pulp regeneration.

## 2. Results

### 2.1. Characterization of Porcine Dental Pulp dECM

**Figure 1:**
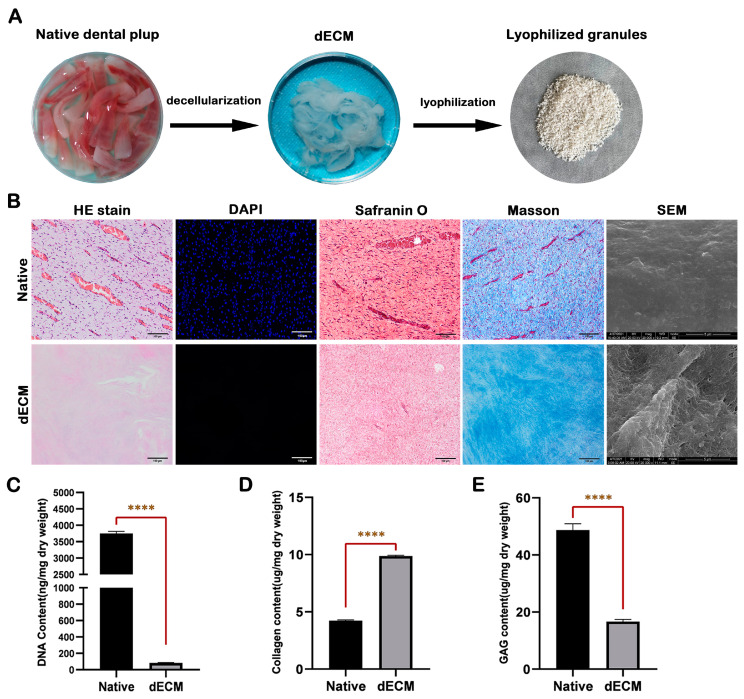
Preparation and characterization of porcine dental pulp dECM. (**A**) Natural dental pulp tissue, dental pulp dECM, and freeze-dried dECM granules. (**B**) H&E, DAPI, Safranin O, Masson’s staining, and SEM analysis were conducted to assess the changes in porcine dental pulp before and after the 12 h decellularization treatment. (**C**) Quantitative analysis of DNA removal (*n* = 3), (**D**) collagen retention (*n* = 3), and (**E**) GAG content in the dECM (*n* = 3). **** *p* < 0.0001.

Following the decellularization process, the previously cellular dental pulp exhibited a notable transformation, appearing white and translucent, with a textured surface, loose consistency, and significantly diminished volume (Figure 1A). Histological (H&E and DAPI) staining revealed that the dECM (12 h treatment group) maintained a relatively intact ECM structure without discernible residual nuclear components (Figure 1B). The residual DNA content was measured at 47.60 ± 2.53 ng/mg (Figure 1C). In contrast, remnants of the nuclear structure were still observable in the 6 h treatment group, with a relatively high residual DNA content of 153.0 ± 9.36 ng/mg (Figure S1). Notably, in the 18 h treatment group, there were no visible remnants of the nuclear components, and the DNA content was merely 36.68 ± 2.48 ng/mg; however, the ECM integrity was severely compromised (Figure S1). Therefore, the 12 h treatment group emerged as the more suitable decellularization regimen for porcine dental pulp. Subsequent Safranin O and Masson’s staining (Figure 1B) and quantitative analysis of collagen (Figure 1D) and Glycosaminoglycan (GAG) (Figure 1E) content provided evidence for the preservation of crucial components within the dECM. Scanning electron microscope (SEM) images showed that compared with the native tissue, although the fibrous network structure in the dECM was exposed, a significant portion of the ECM components was still retained (Figure 1B).

### 2.2. Characterization of the dECM Hydrogels

**Figure 2:**
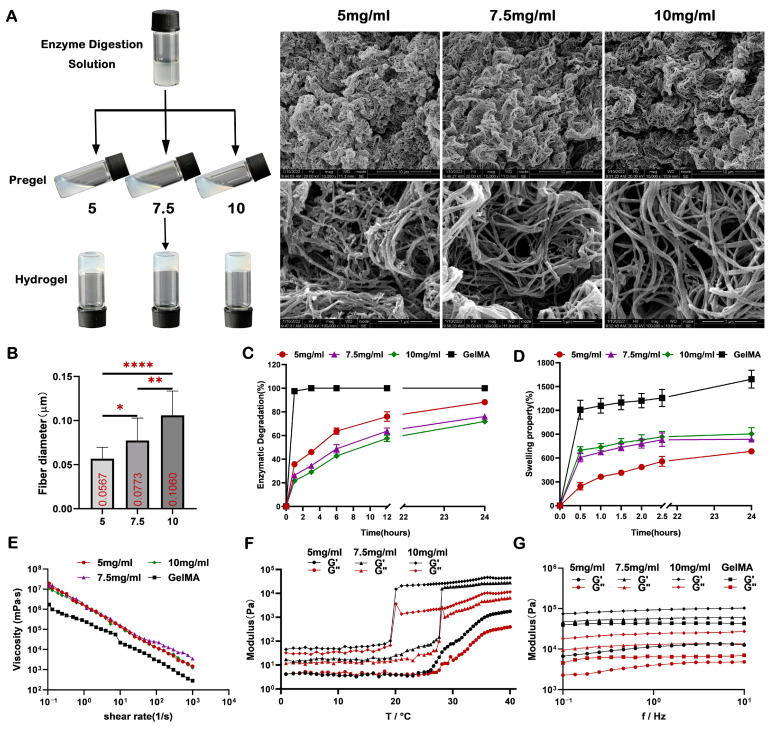
Preparation and characterization of dECM hydrogels with different concentrations. (**A**) neutralization, dilution, gelation, and SEM analysis of dECM hydrogels and (**B**) quantitative assessment of microfiber diameter. (**C**) In vitro enzymatic degradation curves of dECM hydrogels and GelMA hydrogel (*n* = 3). (**D**) Evaluation of the swelling property in PBS (*n* = 4). (**E**) Shear viscosity, (**F**) thermally driven gelation curve, and (**G**) dynamic modulus in rheological analysis (G′: storage modulus, G″: loss modulus). * *p* < 0.05; ** *p* < 0.01; **** *p* < 0.0001.

Before the assessment of the dECM hydrogels, we examined the manipulability of the dECM digestive solution and the gel formation of the dECM pregel solution to determine the appropriate digestion and working concentration. Our findings indicated that when the dissolved concentration exceeded 10 mg/mL, the digestive solution displayed minimal fluidity, hindering subsequent operations. Conversely, when the concentration of the dECM pregel solution dropped below 2.5 mg/mL, it failed to yield a uniform and stable hydrogel structure (Figure S2). Therefore, for subsequent experiments, we utilized 10 mg/mL as the dECM digestion concentration, while 5, 7.5, and 10 mg/mL were chosen as the working concentration for the dECM hydrogel. SEM analysis showed that the dECM hydrogels comprised collagen fibers, with the diameters increasing proportionally with the concentration (Figure 2A,B). Moreover, compared to GelMA hydrogel, the dECM hydrogels exhibited heightened resistance to enzymatic degradation, degrading at a slower rate with increasing concentration (Figure 2C). When immersed in PBS, the dECM hydrogels displayed minimal changes in volume and mass, in stark contrast to the significant expansion observed in the GelMA hydrogel (Figure 2D). This highlights the superior swelling properties of dECM hydrogel, emphasizing its ability to maintain excellent stability in humid environments.

Rheology is commonly employed to characterize the mechanical properties of hydrogels. Biological hydrogel exhibit viscoelasticity, showing both elastic and viscous behavior, thereby manifesting physical properties that lie between those of liquids and solid [52]. The shear viscosity of dECM pregel and GelMA solution decreased linearly within the measured shear rate range (Figure 2E), indicating that both dECM and GelMA hydrogels exhibited shear thinning properties, facilitating their injectability. The kinetics of self-assembly of the dECM hydrogels displayed a substantial increase with the temperature ranging from 0 °C to 40 °C, with higher concentrations leading to lower sol-gel conversion temperatures and shorter sol-gel conversion times (Figure 2F), affirming the temperature sensitivity of the hydrogel. The storage modulus (G′) and loss modulus (G″) of each group of hydrogels (Figure 2G) remained consistent with the increase in frequency, indicating that their elastic solid structures could sustain stability even when subjected to forces of compression and shear during frequency scanning, aligning with the characteristic behavior of biological hydrogel.

### 2.3. dECM Hydrogels Demonstrate Favorable Biocompatibility and Chemotactic Activity, Facilitating Cell Survival, Proliferation, and Migration

**Figure 3:**
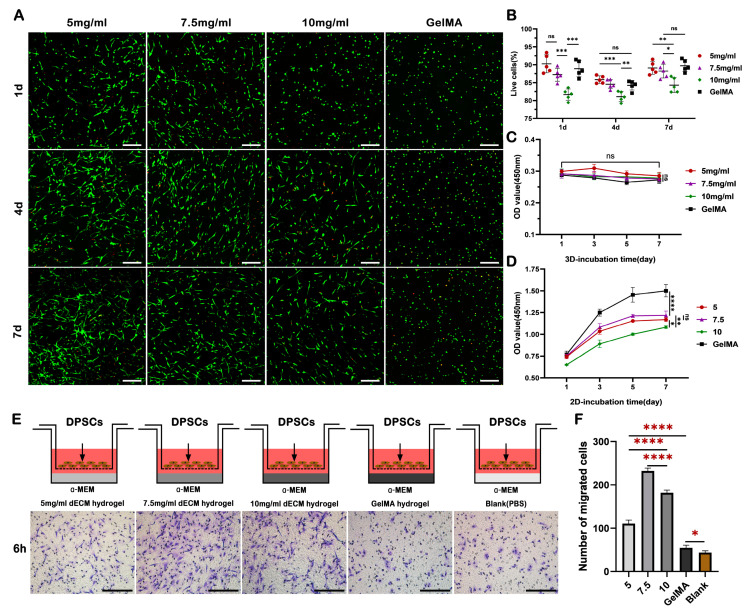
Effects of dECM hydrogels on the survival, proliferation, and migration of DPSCs. (**A**) Live/dead staining and (**B**) cell viability analysis of DPSCs in dECM and GelMA hydrogel over 7 days. (**C**) Assessment of DPSCs proliferation under 3D culture conditions and (**D**) 2D culture conditions, as determined via CCK-8 analysis after 7 days of culture. (**E**) Evaluation of the chemotactic activities of the dECM and GelMA hydrogel diluent on DPSCs by transwell assay. (**F**) Quantitative statistics of the number of DPSCs that migrated to the opposite side of the membrane after 6 h (*n* = 5). Scale bar = 200 μm. * *p* < 0.05, ** *p* < 0.01, *** *p* < 0.001, **** *p* < 0.0001, ns: non-significance.

The cell viability of DPSCs within the hydrogel was assessed at 1, 4, and 7 days of culture. Live/dead staining revealed sustained high cell viability of over 80% in all groups throughout the culture duration, with the 5 mg/mL group exhibiting the most favorable results, albeit without significant disparity when compared to the 7.5 mg/mL and GelMA groups (Figure 3A,B). In addition, compared with the spherical morphology observed in GelMA hydrogel, DPSCs gradually assumed a fusiform shape within the dECM hydrogels (Figure 3A), indicating the superior suitability of dECM hydrogels for promoting DPSCs growth.

CCK-8 analysis was used to evaluate the effect of hydrogels on DPSCs proliferation under both 3D and 2D culture conditions. The findings revealed no discernible proliferation behavior in any of the groups when DPSCs were encapsulated within the hydrogels for 3D culture (Figure 3C). When a 10-fold diluted dECM pregel solution and GelMA solution were employed for the 2D culture, they moderately stimulated cell proliferation; however, the dECM hydrogel groups exhibited lower proliferation rates compared to the GelMA group (Figure 3D). This discrepancy could be attributed to the gradual accumulation and self-assembly of collagen molecules within the dECM pregel solution to form a gel state at 37 °C over the culture period, whereas the GelMA solution remained in a liquid state throughout.

A Transwell assay was conducted to evaluate the chemotactic activity of hydrogels. The results showed that after 6 h of culture, the 7.5 mg/mL group exhibited the highest number of migrated DPSCs to the opposite side of the membrane, followed by the 10 mg/mL group, with the 5 mg/mL group showing the lowest migration rate, albeit significantly higher than that of the GelMA group (Figure 3E,F). This highlights the superiority of dECM hydrogel in promoting cell migration compared to the GelMA hydrogel.

### 2.4. dECM Hydrogels Exhibit Strong Angiogenic Properties and Facilitate DPSCs Differentiation into Odontogenesis and Neurogenesis

**Figure 4:**
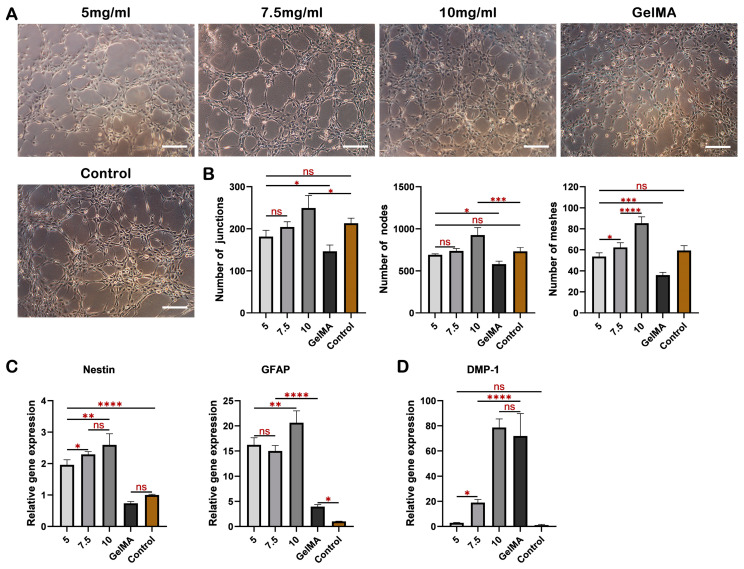
dECM hydrogels promote DPSCs differentiation and tube formation. (**A**) Tube structures formed after 4 h of HUVECs culture with hydrogel extracts in each group. (**B**) Statistical results of tube formation include junctions, nodes, and meshes. Real-time PCR analysis of neurogenic gene expression (**C**) and odontogenic gene expression (**D**) in DPSCs cultured in the four hydrogels for 7 days. Scale bar = 200 μm. * *p* < 0.05, ** *p* < 0.01, *** *p* < 0.001, **** *p* < 0.0001, ns: non-significance.

The angiogenic effects of dECM and GelMA hydrogels on HUVECs were evaluated. After induction for 4 h, all groups successfully developed typical tubular structures, with the exception of the GelMA group (Figure 4A). Within each field, the 10 mg/mL group exhibited a greater number of junctions, nodes, and meshes compared to the other groups, while no significant difference was observed among the 5 mg/mL group, 7.5 mg/mL group, and the control group (Figure 4B).

We conducted real-time PCR analysis to examine the expression of odontogenic and neurogenic genes in DPSCs cultured in the hydrogels for 7 days. The results revealed significantly enhanced expression of the neurogenic genes GFAP and Nestin in the dECM hydrogel groups in comparison to the GelMA group, with the 10 mg/mL group exhibiting the most pronounced effect (Figure 4C). Furthermore, the expression of the odontogenic marker DMP-1 increased proportionally with the concentration within the dECM hydrogel groups, with the 10 mg/mL group demonstrating a level of expression similar to that of the GelMA group (Figure 4D). The high expression of DMP-1 in 10 mg/mL dECM hydrogel and GelMA hydrogel might be attributable to the high matrix stiffness (>30 KPa), known to be more inclined to induce osteogenic differentiation of MSCs [53,54,55,56]. Additionally, functional groups like carboxyl in GeLMA hydrogel have the potential to enrich bioactive ions (such as calcium and/or phosphate ions) from the culture medium to further facilitate the osteogenic differentiation of MSCs [57,58].

### 2.5. dECM Hydrogels Promote Regeneration of Dental Pulp-like Tissue In Vivo

**Figure 5:**
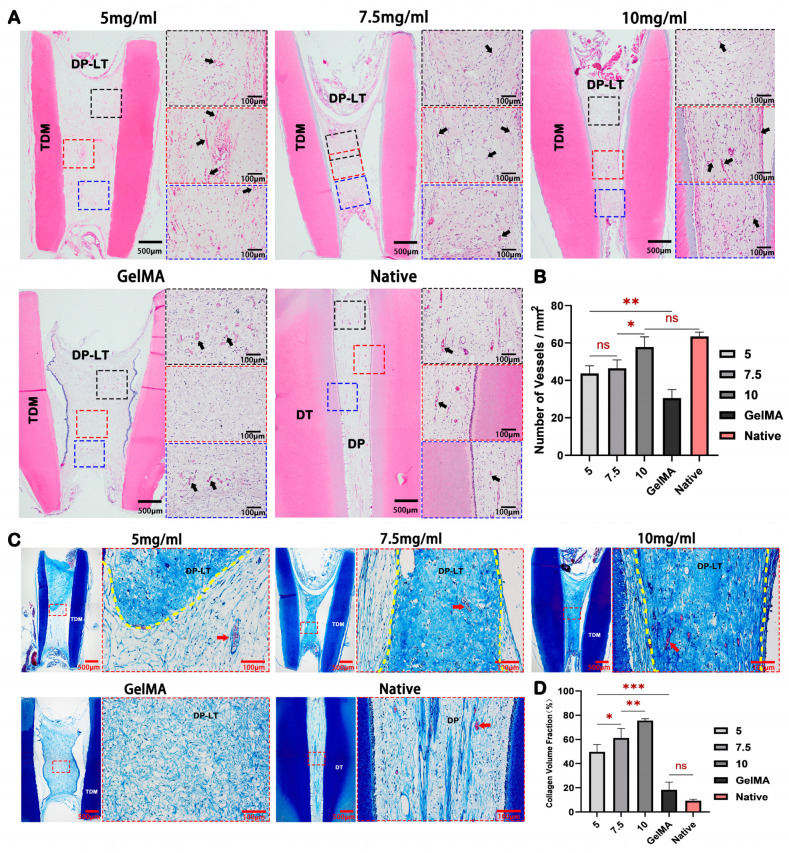
dECM hydrogels promote dental pulp-like tissue regeneration in vivo. (**A**) H&E staining shows that the dental pulp-like tissue rich in blood vessels (black arrows) in the dECM hydrogel groups fills the root canal, while the center of the tissue in the GelMA group lacks blood vessels. (**B**) Semi-quantitative analysis of blood vessel density in the dental pulp-like tissue. (**C**) Masson’s trichrome staining of new dental pulp-like tissue, red arrow: blood vessel, yellow dotted line: dividing line between the modified and unmodified parts of dECM hydrogel. (**D**) Semi-quantitative analysis of collagen volume fraction in new tissue. DT: dentin; TDM: treated dentin matrix; DP: dental pulp; DP-LT: dental pulp-like tissue. * *p* < 0.05, ** *p* < 0.01, *** *p* < 0.001, ns: non-significance.

We employed a nude mouse model to assess the capacity of the hydrogels to induce dental pulp tissue regeneration in vivo. H&E and Masson’s staining showed that the pulp-like tissues in all dECM hydrogel groups exhibited abundant neovascularization throughout the root canals. In contrast, the GelMA group displayed neovascularization solely at both ends of the root canals adjacent to the host tissue, with no neovascularization in the central region of the roots (indicated by black arrows in Figure 5A and red arrows in Figure 5C). This observation underscores the potent pro-angiogenic effect of the dECM hydrogel in vivo. Semi-quantitative analysis of blood vessel density indicated no significant difference between the 10 mg/mL group and the native group (Figure 5B).

Furthermore, we observed that with the region where the remodeling of pulp-like tissue was completed, the collagen fibers in the dECM hydrogel groups exhibited an organized arrangement, particularly in the 7.5 mg/mL group, closely resembling the histological structure of the native group. Conversely, the GelMA group displayed a lack of orderly collagen fiber arrangement (Figure 5C). Semi-quantitative analysis of collagen volume fraction indicated that the dECM hydrogel groups surpassed the native group (Figure 5D). These results suggested that although the dECM hydrogel degrades and remodels slowly in vivo, it can still provide essential fiber components.

**Figure 6:**
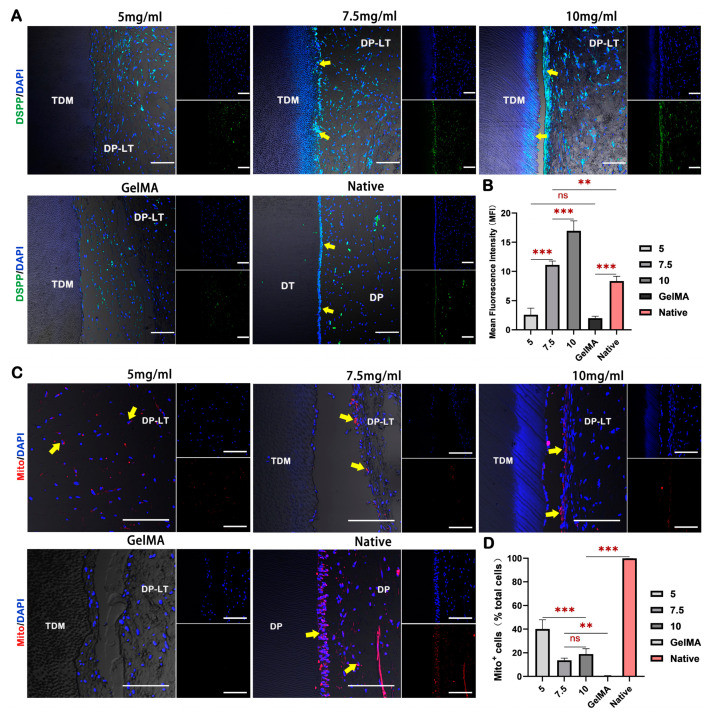
dECM hydrogels promote the formation of pulp–dentin complex-like structures and support the survival of transplanted DPSCs. (**A**) IF staining of DSPP in dental pulp-like tissue, DSPP: green; yellow arrow: odontoblast-like cells. (**B**) Semi-quantitative analysis of IF intensity of DSPP expression. (**C**) IF staining of human cell-specific protein mitochondria (Mito) in dental pulp-like tissue, Mito: red; yellow arrow: Mito-positive cells. (**D**) Quantitative analysis of the proportion of Mito-positive cells. DAPI: blue, cell nucleus; DT: dentin; TDM: treated dentin matrix; DP: dental pulp; DP-LT: dental pulp-like tissue; scale bar = 100 μm. ** *p* < 0.01, *** *p* < 0.001, ns: non-significance.

Additionally, immunofluorescence (IF) staining was used to identify the newly formed pulp–dentin complex. The results revealed the presence of a layer of odontoblast-like cells with heightened expression of the odontoblast protein DSPP in the 7.5 and 10 mg/mL groups, located in close proximity to the dentin tubule wall, with visible cell protrusions extending into the dentin tubules, mirroring the corresponding structure observed in the native group. Conversely, no similar observable phenomenon was noted in the 5 mg/mL and GelMA groups (Figure 6A,B).

Finally, IF staining of human cell-specific protein mitochondria was conducted to assess the hydrogels’ capacity to sustain the viability of transplanted DPSCs in vivo. The findings demonstrated that all dECM hydrogel groups effectively maintained the viability of the transplanted human DPSCs for a duration of 8 weeks. In contrast, the transplanted DPSCs in the GelMA group exhibited minimal survival (Figure 6C,D). The sustained survival of DPSCs further implies that transplanted stem cells may play a direct role in the regeneration of dental pulp. Overall, the dECM hydrogel proves to be more suitable as a scaffold material for pulp regeneration than the GelMA hydrogel, with the 7.5 mg/mL concentration emerging as the most optimal for dental pulp regeneration using the dECM hydrogel alone.

## 3. Discussion

Despite significant advancements in dental pulp tissue engineering over the last decade, a universally recognized scaffold material optimally suited for functional dental pulp regeneration remains elusive [59]. Our research is dedicated to the quest for such specialized scaffold materials, wherein the utilization of dECM materials based on decellularization technology has captured our interest. These materials preserve vital tissue-specific biochemical cues and extracellular matrix (ECM) functions, essential for successful regeneration [23,24,60]. Furthermore, dECM hydrogels derived from natural organs or tissues exhibit structural properties and biological hydrogel reactivity, such as fluidity and thermal responsiveness [61]. These attributes align with the requirements of dental pulp tissue engineering scaffold materials, particularly in accommodating the intricate root canal system, thereby serving as our impetus for the development of pulp tissue-specific dECM hydrogels.

The initial step in the production of the dECM hydrogel involves the decellularization of the dental pulp tissue. Sodium dodecyl sulfate (SDS) and Triton X-100 are commonly employed detergents in tissue decellularization processes [62], with SDS being particularly prevalent in the decellularization of porcine dental pulp [48,50,51]. However, in this study, considering the dominance of type I collagen in dental pulp matrix composition [51], we intentionally avoided the use of SDS in our optimal protocols for porcine pulp tissue decellularization. This decision was influenced by the potential drawbacks of SDS despite its potent decellularization efficacy. SDS has been associated with collagen denaturation and the exposure of concealed antigens within it, which could potentially trigger a host immune response against the graft [62,63]. Additionally, the ionic nature of SDS makes its complete removal challenging, leading to residual SDS in the decellularized extracellular matrix (ECM), resulting in persistent cytotoxicity and inflammatory reactions in the transplanted area [64,65]. Conversely, the non-ionic detergent TritonX-100 acts by disrupting DNA–protein, lipid–lipid, and lipid–protein interactions to eliminate cellular components without compromising natural protein structures [60]. Tissues treated with Triton X-100 display relatively low cytotoxicity due to its ease of cleansing [62]. Hence, considering its gentle yet effective decellularization properties, using Triton X-100 instead of SDS for collagen-rich tissue decellularization appears justifiable. Notably, a study optimizing bovine dental pulp decellularization demonstrated that Triton X-100 treatment minimally affected the organic matrix and ultrastructure of dental pulp tissue [66]. Building upon this method and considering the similarities and differences in dental pulp tissue composition among various species [67], we compared three distinct decellularization protocols and ultimately selected the most optimal one based on the effect of decellularization. Furthermore, the current international standards for assessing the efficacy of decellularization primarily stipulate that the residual double-stranded DNA content should be below 50 ng/mg (dry weight), the DNA fragment length should be less than 200 bp, and there should be no nuclear matter visible in the dECM via DAPI or H&E staining [62]. Accordingly, our study employed histological staining and DNA content analysis to validate the decellularization effects of each protocol. The findings revealed that the 12 h group incurred minimal damage to the porcine dental pulp ECM while ensuring complete removal of DNA components (Figure S1). Subsequent identification confirmed that the dECM prepared by the 12 h group retained a substantial portion of collagen components, thereby facilitating the subsequent preparation of the dECM hydrogel.

Following successful decellularization, converting the dECM from its solid state into an injectable hydrogel becomes essential, enabling the convenient filling of irregularly shaped spaces in the root canal [68]. It is widely acknowledged within the research community that collagen inherently possesses self-assembly properties. Collagen self-assembly follows a two-stage kinetic process involving the formation of assembly nuclei and their continuous growth, influenced by several factors like solution pH, temperature, concentration, and ionic strength [69,70,71]. It is precisely via this fundamental mechanism that the solid form of dECM materials can be converted into hydrogel form after digestion and dissolution into soluble collagen components [61]. Numerous studies have demonstrated that most dECM collagen molecules derived from natural tissues can self-assemble under suitable pH conditions of 7.2–7.4 and physiological temperatures [32,69,70]. Similarly, our investigation unveiled that porcine dental pulp dECM hydrogels can also undergo sol-gel conversion at neutral conditions and physiological temperatures without requiring additional cross-linking agents. Additionally, the self-assembly of collagen exhibits a concentration-dependent threshold. When the collagen concentration exceeds a certain threshold, visible aggregation occurs; conversely, concentrations below the threshold do not exhibit evident self-assembly behavior [71,72]. Thus, in this study, we initially explored the concentration threshold of porcine dental pulp dECM hydrogel. Results indicated that a minimum concentration of 2.5 mg/mL was necessary to form internally stable cross-linked aggregates. Biological hydrogels should generally display viscoelastic characteristics between liquids and solids to mimic the physical parameters of natural ECM [52,55]. In our investigation, the results from frequency sweeps indicated that the storage modulus (G′) consistently surpassed the loss modulus (G″) and exhibited stability with escalating frequencies, confirming the inherent viscoelastic properties typical of the dECM hydrogels. Additionally, rheological viscosity tests underscored the favorable injectability of the dECM pregel solution prior to gelation, demonstrating its remarkable capability to conform to the intricate structures of the root canal system.

A previous study evidenced the capacity of human dental pulp dECM hydrogel coatings to stimulate migration and proliferation and induce multilineage differentiation of DPSCs [32]. It is important to recognize that DPSCs naturally reside within the 3D microenvironment of the natural dental pulp, significantly distinct from the coatings. Therefore, there is a paramount need to focus efforts on cultivating stem cells in a 3D manner to effectively replicate their natural in vivo surroundings [73]. In pursuit of this objective, we employed a 3D culture model to assess the impact of porcine pulp dECM hydrogels on human DPSCs behaviors. Initial results from live/dead staining demonstrated the excellent biocompatibility of dECM hydrogels, facilitating DPSC spreading and extension. This positive effect can be attributed to the abundance of active ECM components and cell adhesion sites present within the dECM hydrogels [24]. However, unlike the observed proliferation of DPSCs on dental pulp dECM hydrogel coatings [32], the DPSCs encapsulated in GelMA and dECM hydrogels did not exhibit noticeable proliferation behavior. Interestingly, upon reverting to a 2D culture environment, DPSCs demonstrated a certain level of proliferation. This could be attributed to the potential limitation on DPSC proliferation imposed by the high stiffness of the matrix. Revital Goldshmid et al. highlighted an inverse relationship between the cell proliferation rate and the shear storage modulus of hydrogels; compared to high proliferation rates (87–91%) in low modulus PEG-fibrinogen hydrogels (G′ = 250 Pa), proliferation rates in high modulus materials (G′ = 2000 Pa) were notably lower, ranging from 33% to 42% [74]. In our study, the storage modulus (G′) of each group of hydrogels exceeded 5000 Pa, significantly inhibiting DPSCs proliferation. Furthermore, compared to GelMA hydrogel, the dECM hydrogels exhibited a notable capacity to promote cell migration. This aligns with earlier findings indicating that dental pulp ECM components possess robust recruitment capabilities for DPSCs [75], suggesting the potential utility of the dECM hydrogel in cell-free strategies for pulp tissue engineering [76].

The native dental pulp tissue can be broadly categorized into four layers: the odontoblast layer close to the dentin, the underlying acellular layer, the multicellular layer internally, and the central area comprising the intrinsic dental pulp rich in blood vessels and nerves, serving functions such as dentin formation, nutrition, sensation, defense, and repair [15]. Therefore, the concept of ideal pulp regeneration involves the precise regeneration of natural structures, including the pulp–dentin complex, abundant blood vessels, and nerve fibers, ultimately achieving the restoration of physiological functions such as nutrition, dentin formation, sensation, and immunological defense [13]. Angiogenesis plays a pivotal role in providing nutrition and oxygen supply and is considered a crucial factor for successful dental pulp tissue engineering [77,78]. In our study, compared to GelMA hydrogel, dECM hydrogels exhibited a robust capacity to stimulate angiogenesis both in vitro and in vivo, likely attributable to the retention of a substantial amount of VEGF within the dECM materials derived from dental pulp tissue [49,79]. We believe that this proangiogenic ability significantly sustains the activity of transplanted stem cells in vivo. As evidenced by the specific human cell protein mitochondria IF staining, the dECM hydrogels could sustain the activity of DPSCs for up to 8 weeks. Additionally, the pulp–dentin complex represents a specialized structure within dental pulp tissue, serving the function of dentin formation and repair [3]. Two odontoblast proteins, DMP-1 and DSPP, are recognized as positive regulators of hard tissue mineralization [80], showing high expression in odontoblasts and participating in dentinogenesis and pulpal repair [81,82]. In our study, it was exciting to note that 7.5 and 10 mg/mL dECM hydrogels not only significantly induced expression of DMP-1 in DPSCs in vitro but also facilitated the regeneration of a continuous odontoblast-like cell layer with high DSPP expression near the root canal wall in vivo and closely resembled the corresponding structure of the native dental pulp. The continuous odontoblast-like cell layer is rare in other hydrogels used for pulp regeneration, such as self-assembled peptides [83] and collagen gel [84]. This may be related to the fact that dental pulp dECM hydrogel can provide complex and comprehensive regeneration signals, which are unmatched by any other hydrogels. In contrast, while GelMA hydrogel could induce high DMP-1 expression in DPSCs in vitro, it failed to regenerate an odontoblast-like cell layer in vivo. This discrepancy could be attributed to the incapacity of GelMA hydrogel alone to induce rapid angiogenesis, thereby significantly reducing the survival rate of transplanted DPSCs and weakening their regenerative effects. Finally, the evaluation of neurogenesis represents an integral component of comprehensive dental pulp regeneration accompanied by multifunctional recovery [3]. In our study, the capacity of each group of dECM hydrogels to promote the expression of neurogenesis-related markers Nestin and GFAP in DPSCs surpassed that of GelMA hydrogels, once again underscoring the abundance of neurogenesis-participating proteins within dental pulp dECM hydrogels [32]. These cumulative findings strongly suggest that porcine dental pulp dECM hydrogel holds immense potential in achieving vascularization, neurogenesis, and pulp–dentin complex formation, essential components in dental pulp regeneration. Compared to GelMA hydrogel, dental pulp dECM hydrogel can serve as tissue-specific scaffold materials for dental pulp regeneration.

Nevertheless, in this study, disparities exist between the subcutaneous ectopic environment on the backs of nude mice and the in situ environment within the root canals of the jaws, necessitating subsequent in situ experiments on larger animals to comprehensively evaluate the impact of this hydrogel on dental pulp regeneration. Furthermore, the correlation between the degradation or remodeling rate of dECM hydrogels and the tissue regeneration rate remains an aspect warranting further investigation. Research has indicated that the degradation rate of dECM materials can be regulated via the addition of specific biological compounds or chemical modifications [85,86]. Conversely, the notable combination of high osteogenic activity and a slow degradation rate observed in the high-concentration dECM hydrogel implies its potential as a scaffold for bone tissue regeneration. Its application might be further extended by tailored modifications in specific directions in future studies.

## 4. Materials and Methods

### 4.1. Decellularization of Porcine Dental Pulp

The porcine mandibles aged from 8 months to 1 year old were purchased in the slaughterhouse (Chengdu, China); pulp tissues were excised from the permanent incisor germs and cut into strips, rinsed in sterile phosphate-buffered saline (PBS) 3 times, followed by rinsing with double deionized water 3 times. Three protocols were established for decellularization of porcine dental pulp to select the optimal one: the samples were firstly treated with 0.02% trypsin (Hyclone, Logan, UT, USA) and 0.05% ethylenediaminetetraacetic acid (EDTA) at 37 °C for 6 h, 12 h, and 18 h, respectively, followed by 2% Triton X-100 (Sigma, St. Louis, MO, USA) at 4 °C for 3 h, and finally treated with 0.02 mg/mL DNase I (Sigma, St. Louis, MO, USA) at 37 °C for 24 h. All reagents were prepared with double deionized water, all steps were carried out on a shaker and rinsed with double deionized water at 4 °C for 12 h between every two steps, and the water was changed every 3 h. Finally, the samples were sterilized by soaking in 100 U/mL penicillin and 100 mg/mL streptomycin (Hyclone, Logan, UT, USA) for 24 h, rinsed with double deionized water for 12 h, lyophilized for 8 h, and stored at −20 °C for further use.

### 4.2. Characterization of Porcine Dental Pulp dECM

Native and decellularized dental pulp were fixed in 4% paraformaldehyde for 12 h at room temperature. The surface morphology of the samples was analyzed with SEM (Inspect F, FEI, Hillsboro, OR, USA). Subsequently, samples were dehydrated in a graded ethanol series, embedded in paraffin, and dissected into 5 μm sections. The tissue slides were stained with hematoxylin and eosin (H&E), 4′,6-diamidino-2-phenylindole (DAPI), Masson’s Trichrome (Baso, Shanghai, China), and Safranin O (Solarbio, Beijing, China) to evaluate the cellular removal, collagen distribution, and glycosaminoglycan (GAG) retention, respectively. The amount of double-stranded DNA was quantified using a DNA extraction kit (TIANGEN, Beijing, China). The DNA concentration was determined using a Nanodrop instrument (Thermo Fisher Scientific, Waltham, MA, USA). Collagen and GAG contents were quantitated using a hydroxyproline assay kit (Solarbio, China) and a Blyscan™ Sulfated GAG assay kit (Biocolor, Carrickfergus, UK), respectively. All procedures were performed according to the manufacturer’s protocols.

### 4.3. Fabrication of dECM Hydrogels

To evaluate the optimal dissolution concentration, the freeze-dried dECM particles were first digested in 0.5 mol/L acetic acid containing pepsin at three concentrations of 10, 15, and 20 mg/mL, respectively, for 72 h on a magnetic agitator at room temperature (dECM/pepsin = 10/1, *w*/*w*), and then the undissolved particulates were removed by centrifugation at 2000 rpm for 10 min at room temperature. Then, the solution pH was adjusted to neutral by dropwise addition of precooled 10 mol/L NaOH, followed by cautiously adding one-ninth of the final volume of 10× PBS to adjust the physiological acidity and salinity. Finally, the pregel solution was diluted into 5 concentrations of 1, 2.5, 5, 7.5, and 10 mg/mL to screen the appropriate working concentration with PBS and gelled at 37 °C for 30 min when used. The dECM hydrogels were used at concentrations of 5, 7.5, and 10 mg/mL in the following experiments. These above steps were carried out on the ice to prevent the solution from gelling in advance. The dECM pregel solution can be stored at −20 °C for at least one month, slowly thawed on the ice for 8 h before use and cannot be frozen and thawed repeatedly.

As a control, a 5% (*w*/*v*) GelMA solution was created by dissolving lyophilized GelMA (EFL-GM-60, Engineering for Life, EFL, Suzhou, China) in sterile PBS containing 0.5% (*w*/*v*) lithium phenyl-2,4,6-trimethylbenzoylphosphinate (LAP, Engineering for Life, EFL, Suzhou, China). Blue light irradiation for 30 s formed a stable GelMA hydrogel.

### 4.4. SEM Analysis

The microstructure of the dECM hydrogels was evaluated via SEM. dECM hydrogels were fixed in 2.5% (*w*/*v*) glutaraldehyde overnight and dehydrated in ascending ethanol concentrations. Samples were then exposed to 100% hexamethyldisilazane solution and left for evaporation overnight, gold sputter coated, and viewed using a scanning electron microscope (Inspect F, FEI, Hillsboro, OR, USA).

### 4.5. Rheological Characterization

The rheological properties of dECM hydrogels and GelMA hydrogel were analyzed using an HAAKE Viscotester iQ Air (Thermo Scientific, Waltham, MA, USA). Shear rate sweeps were performed using the C35 1°/Ti cone rotator with a truncation gap distance of 1 mm to determine the shear viscosity of samples by changing the applied shear rate from 0.1 to 1000 s^−1^ at 15 °C. Small amplitude oscillatory shear (SAOS) tests at a strain amplitude of 1% and a frequency of 1 Hz, while the temperature increased from 0 °C to 40 °C at a rate of 1 °C min^−1^, were investigated using the parallel rotator to determine the thermally driven gelation of the dECM samples. Frequency sweeps were conducted using the parallel rotator to characterize the dynamic modulus of the samples after gelation. The applied strain constant was determined to be 1% over the frequency range of 0.1–10 Hz at 37 °C.

### 4.6. Swelling and Degradation Analysis In Vitro

The swelling property of dECM and GelMA hydrogels was investigated via the conventional gravimetric method [87]. Briefly, the lyophilized samples were immersed in PBS at 37 °C for predetermined time intervals (0.5, 1, 1.5, 2, 2.5, and 24 h, *n* = 4). The initial weight of the dry samples before incubation was recorded as W0. At designated time points, the samples were taken out of PBS to measure the weight, recorded as W1. Prior to weighing, the hydrogel was placed between two filter papers to gently remove excess liquid. Then, the hydrogels were placed back into the PBS. The swelling rate was calculated as the following equation: Swelling property (%) = (W1 − W0) × 100/W0.

The enzymatic degradation rate of samples was determined with reference to the previously described [88]. Briefly, lyophilized samples were weighed (W0) and incubated in PBS supplemented with 0.5 mg/mL of collagenase type I (Sigma-Aldrich, St. Louis, MO, USA) at 37 °C, and at determined time points (1, 3, 6, 12, and 24 h, *n* = 3), samples were picked out for lyophilization and weighed (W1). The degradation rate was calculated using the equation Enzymatic degradation (%) = (W0 − W1) × 100/W0.

### 4.7. Cell Isolation and Culture

The human dental pulp and root were obtained from the impacted third molars or premolars of 12- to 20-year-old young, healthy patients whose teeth were extracted for clinical reasons, which were collected from the West China Hospital of Stomatology, Chengdu, China. All experiments were conducted with ethical approval from the Committee of Ethics of Sichuan University, and written informed consent was obtained from all guardians on behalf of the children and teenagers enrolled in this study. The approval number is WCHSIRB-CT-2022-194. DPSCs were isolated and cultured as described previously [89]. Briefly, dental pulp tissue was scissored into tiny pieces of about 1 mm × 1 mm before being digested with 3 mg/mL collagenase type I (Sigma-Aldrich, St. Louis, USA) for 30 min at 37 °C. The digested tissue and single cells were incubated in α-MEM supplemented with 10% fetal bovine serum (FBS, HyClone, Logan, USA) and 1% penicillin–streptomycin in a humidified 5% CO_2_ atmosphere at 37 °C. DPSCs were used in passages 3–5 in the following experiments. HUVECs (iCell Bioscience Inc., Shanghai, China) were cultured in endothelial cell medium (ScienCell, San Diego, CA, USA) at 37 °C with 5% CO_2_. HUVECs were used in passages 4–7. The culture medium was changed every 2 days.

### 4.8. Cell Proliferation

Under 2D and 3D culture conditions, the effects of GelMA and dECM hydrogels on the proliferation of DPSCs were detected using the cell counting kit-8 (CCK-8, Key Gen, Nanjing, China) after 1, 3, 5, and 7 days of culture, and the optical density (OD) values at 450 nm were determined using a spectrophotometer (Thermo Fisher Scientific, Waltham, USA). In the 3D culture, DPSCs were mixed with the GelMA solution and dECM pregel solutions at the density of 2 × 10^5^ cells/mL, and the solutions were then inoculated into a 96-well plate with 50 μL/well. After gelation, respectively, 100 μL complete culture medium (α-MEM supplemented with 10% FBS and 1% penicillin–streptomycin) was added to each well. In 2D culture, DPSCs were inoculated in a 96-well plate with a density of 1 × 10^4^ cells/well. After cell adhesion, 100 μL complete culture medium containing 0.5, 0.75, and 1 mg/mL dECM pregel and 0.5% GelMA, respectively, were added to the corresponding wells. The culture medium was changed every 2 days.

### 4.9. Cell Viability

Under the 3D culture condition, according to the manufacturer’s instructions, a Live/Dead Viability/Cytotoxicity kit (Key Gen, Nanjing, China) was used based on fluorescence observation to evaluate the cell viability. DPSCs were mixed with different concentrations of dECM pregel solutions and GelMA solution at the density of 2 × 10^6^ cells/ml, and then the suspensions were inoculated into a 15 mm Petri dish with 200 μL/dish, gelled, respectively, and cultured in an incubator at 37 °C. After culturing for 1, 4, and 7 days, five randomized images from each group were captured and imported into ImageJ software Version 1.54f for cell counting.

### 4.10. Cell Migration

DPSC migration was measured using Transwell assays by using a Chemotaxicell chamber (8 μm pore size, Corning, New York, NY, USA), briefly, 200 μL of dECM pregel solutions (5, 7.5, and 10 mg/mL) and GelMA solution were separately added to the lower chamber while PBS was used as the blank control and 300 μL complete culture medium was added after gelation, respectively. After serum starvation for 24 h, 5 × 10^4^ DPSCs were seeded to the upper chamber and incubated at 37 °C for 6 h. Cells that migrated to the lower surface of the membrane were fixed with 4% paraformaldehyde and stained with 1% crystal violet; images were obtained using an inverted microscope.

### 4.11. Tube Formation

Tube formation assays were performed using Matrigel (Corning, New York, USA). Briefly, 4 mL endothelial cell medium was added to 1 mL dECM hydrogels (5, 7.5, and 10 mg/mL) and GelMA hydrogel and soaked at 37 °C for 24 h to prepare conditional medium. Then, 1 × 10^5^ HUVECs were seeded into 15 mm Petri dishes coated with Matrigel and cultured with various conditional media for 4 h. Endothelial cell medium served as the control group. Phase-contrast images were acquired using an inverted microscope (Olympus, Tokyo, Japan). The numbers of nodes, junctions, and meshes of tubes in each field were determined with Image J software.

### 4.12. Odontogenic and Neurogenic Differentiation

DPSCs were mixed with different dECM pregel solutions (5, 7.5, and 10 mg/mL) and GelMA solution at the density of 2 × 10^6^ cells/mL, and then the various suspensions were inoculated into a 24-well plate with 100 μL/well, gelled, respectively, and cultured in an incubator at 37 °C. The expression of the odontogenic gene DMP-1 and neurogenic genes GFAP and Nestin were analyzed using real-time PCR after 7 days of culture. The primer sequences are listed in Table S1. Relative expression levels were calculated using the 2^−ΔΔCT^ method and normalized to the reference h-GAPDH gene.

### 4.13. Preparation of Treated Dentin Matrix (TDM)

TDM can release a variety of dentinogenic factors and proteins, including DMP-1, DSPP, TGF-β1, decorin, COL-1, Biglycan, etc., and can induce and support the regeneration of complete dentin tissues [90], commonly used as a peripheral biological scaffold for in vivo dental pulp regeneration to simulate the environment inside the root canal. TDM was prepared as previously described [90]. Briefly, by grinding along the tooth profile, the periodontal tissues, outer cementum, and part of the dentin of human premolars were removed. The crown and root apex were cut off, retaining the middle part of the root, and grinding it into a root segment about 5 mm long. Dental pulp tissues and pre-dentin were mostly removed using mechanical means. The resulting dentin matrix was soaked in deionized water and mechanically cleaned for 20 min, soaked in 17%, 10%, and 5% EDTA (Sigma, St. Louis, USA) for 5 min in sequence, and washed in deionized water for 10 min in an ultrasonic cleaner during the interval. The resulting TDM was then maintained in sterile PBS with 100 units/mL penicillin and 100 mg/mL streptomycin (Hyclone, Logan, USA) for 72 h, and washed in sterile deionized water for 10 min in an ultrasonic cleaner, and finally stored at 4 °C for later use.

### 4.14. Animal Studies

All animal experiments in this study were conducted in accordance with the guidelines of the Ethics Committee of West China Hospital of Stomatology, Sichuan University (approval number: WCHSIRB-D-2022-319; approval time: 5 May 2022). Ten female nude mice (8 weeks old) were obtained from DS Experimental Animals Co., Ltd. (Chengdu, China).

DPSCs were mixed with dECM pregel solutions and GelMA solution at the density of 2 × 10^6^ cells/ml, and then the suspension was injected into the TDM tubes. After gelation, respectively, TDM tube samples were transplanted subcutaneously into the back of nude mice. The experiment was divided into four groups: (1) 5 mg/mL dECM hydrogel, (2) 7.5 mg/mL dECM hydrogel, (3) 10 mg/mL dECM hydrogel, and (4) GelMA hydrogel. Each mouse was implanted with four samples, one from each group. The animals were sacrificed after 8 weeks, and the grafts were harvested.

### 4.15. Histological Analysis

The harvested grafts were fixed with 4% paraformaldehyde, decalcified with 10% buffered EDTA for 3 months, and paraffin-embedded and sectioned further into 5 μm thick slices. H&E (Solarbio, Beijing, China) and Masson’s trichrome (Baso, Shanghai, China) staining were performed according to the manufacturer’s recommended protocols to observe the structure and angiogenesis of regenerated dental pulp-like tissue. Immunofluorescent (IF) staining was used to analyze the formation of the odontoblast layer and the retention of transplanted DPSCs in regenerated dental pulp-like tissue. The primary antibodies used for IF included anti-DSPP (1:200 dilution, Abcam, Cambridge, UK) and anti-mitochondria (1:400 dilution, Abcam, Cambridge, UK). Alexa Fluor 488-conjugated Goat anti-rabbit, and Alexa Fluor 555-conjugated goat anti-mouse (1:200 dilution, Invitrogen, Carlsbad, CA, USA) secondary antibodies were used. Sections and cells were mounted in a fluorescent mounting medium after DAPI counterstaining and were examined under a laser scanning confocal microscope (Olympus, Tokyo, Japan).

### 4.16. Statistical Analysis

All experiments in this study were performed three times. Statistical analysis was performed using GraphPad Prism software Version 8.0.2 (GraphPad Software, San Diego, USA), and all data were expressed as mean ± standard deviation (mean ± SD). Significant differences were assessed by One-way ANOVA, Two-way ANOVA, or Student’s *t* test. *p* < 0.05 *, *p* < 0.01 **, *p* < 0.001 ***, and *p* < 0.0001 **** were considered to be statistically significant, and ns represented non-significance.

## 5. Conclusions

In this study, we successfully developed a porcine dental pulp dECM hydrogel exhibiting outstanding rheological and biological properties. The dECM hydrogel demonstrated the ability to provide crucial biological cues, effectively inducing odontogenic and neurogenic differentiation of DPSCs in vitro. Moreover, it exhibited remarkable angiogenic potential, contributing to the successful regeneration of dental pulp-like tissue characterized by a robust vascular network and odontoblast-like cell layer in vivo. In summary, the porcine dental pulp dECM hydrogel presents itself as a promising tissue-specific scaffold material for facilitating functional dental pulp regeneration, thus holding significant potential for broad clinical application.

## Data Availability

The data are not publicly available due to privacy or ethical restrictions.

## Supplementary Materials

The following supporting information can be downloaded at https://www.mdpi.com/article/10.3390/ijms242417483/s1.
