# Supplementary material for: Injectable Xenogeneic Dental Pulp Decellularized Extracellular Matrix Hydrogel Promotes Functional Dental Pulp Regeneration
Source: Int J Mol Sci. 2023 Dec 14;24(24):17483. doi: 10.3390/ijms242417483 (PMC10743504; doi:10.3390/ijms242417483)
Supplement: Supplementary file 1 [file ijms-24-17483-s001.zip › ijms-2735658-supplementary.pdf]

Supplementary information and results

Table S1. Quantitative Real-time Polymerase Chain Reaction Primer Information

Gene	Prime sequence (F, forward; R, reverse; 5′ to 3′)
DMP-1	F: CTCGCACACACTCTCCCACTCAAA
	R: TGGCTTTCCTCGCTCTGACTCTCT
GFAP	F: AGGACCTGCTCAATGTCAAG
	R: GAATGGTGATCCGGTTCTCC
Nestin	F: AGGAATGCCGCTAGTCTCTGA
	R: GGACTCTCTATCTCCTTCCCTCTG
GAPDH	F: CCTGGGCTACACTGAGGACC
	R: CATACCAGGAAATGAGCTTCAC

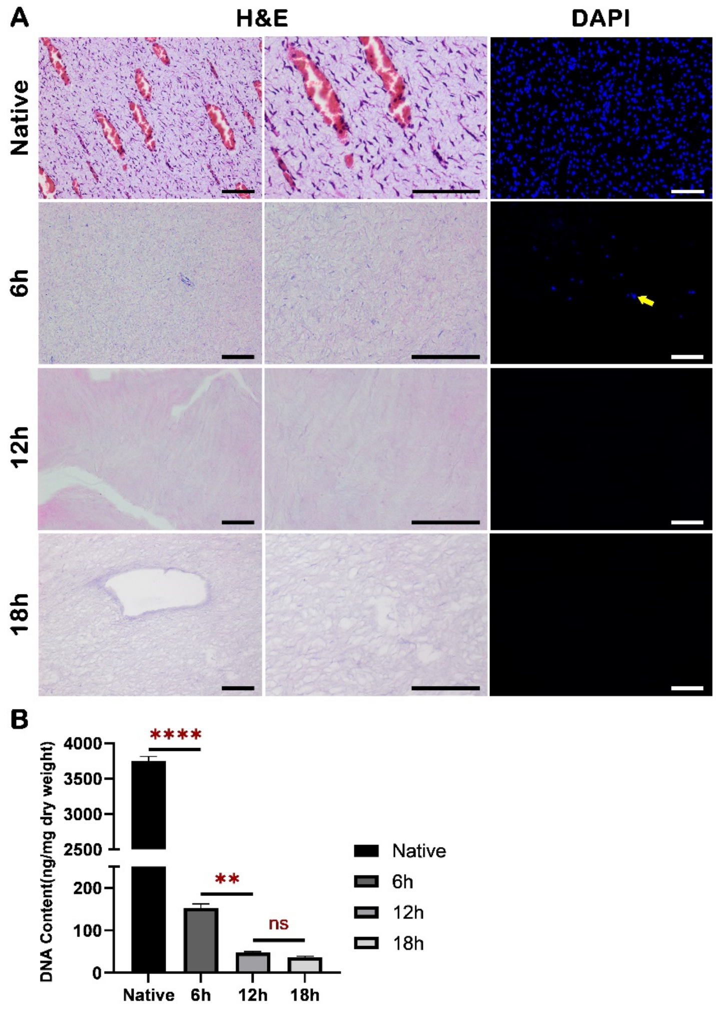

Figure S1. Exploration of decellularization protocol for porcine dental pulp tissue.

(A) H&E and DAPI staining of porcine dental pulp dECM obtained by different decellularization protocols; blue: cell nuclei, yellow arrow: remaining cell nuclei after decellularization. (B) Quantification of residual DNA content. Scale bar = 100 μm. **p < 0.01, ****p < 0.0001, ns: non-significance.

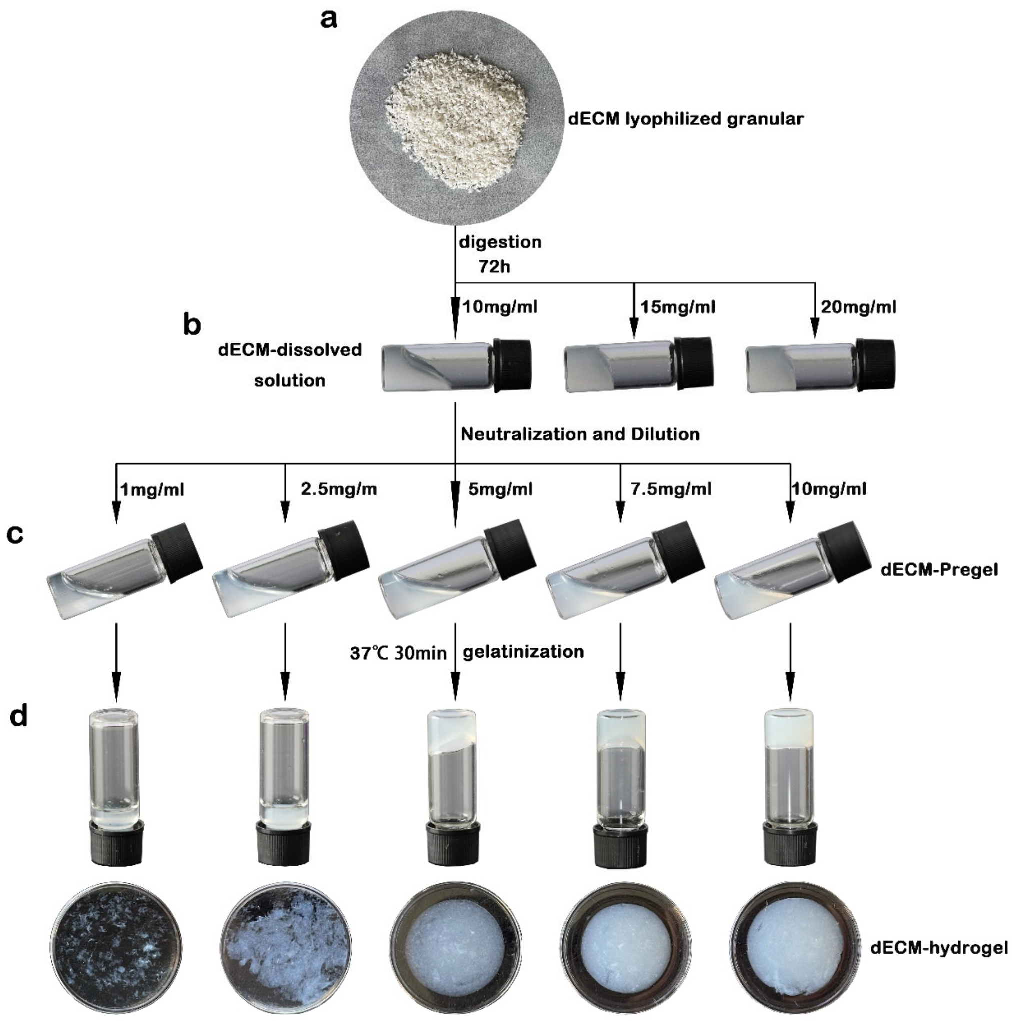

Figure S2. Exploration the appropriate digestion and working concentration of dECM hydrogels.
(**a**) dECM lyophilized particles. (**b**) Fluidity of dECM digestion solutions with different concentrations after the glass bottles were placed horizontally for 15 minutes. (**c**) dECM pregel solutions with different concentrations. (**d**)Gelation state of dECM hydrogels with different concentrations after 30 minutes at 37°C.
